# Supplementary material for: Identification of Clinical Value and Biological Effects of XIRP2 Mutation in Hepatocellular Carcinoma
Source: Biology (Basel). 2024 Aug 19;13(8):633. doi: 10.3390/biology13080633 (PMC11351838; doi:10.3390/biology13080633)
Supplement: Supplementary file 1 [file biology-13-00633-s001.zip › biology-3086203-supplementary.pdf]

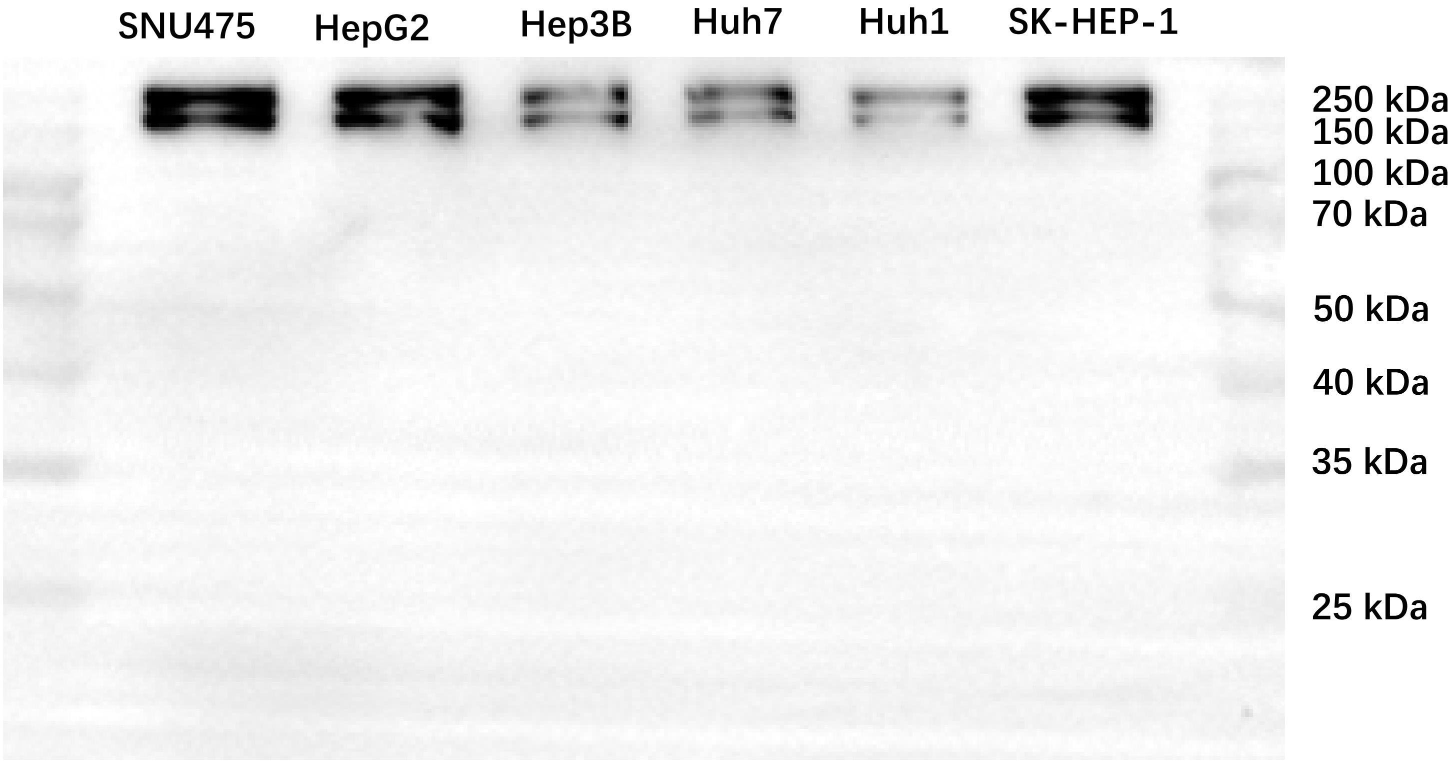

Figure S1 Western blot membrane of XIRP2 (>250 kDa) protein detected with anti-XIRP2 (1:500; cat no. 11896-1-AP, Proteintech, Wuhan, China) antibody. Gel-separated proteins were transferred to nitrocellulose membranes (0.2 um pore size; Thermo Fisher Scientific, USA) by wet electroblotting (310 mA, 120 min). Membranes, incubated with a HRP-conjugated Affinipure Goat Anti-Rabbit IgG(H+L) (1:3000, Cat no. SA00001-2, Proteintech, Wuhan, China), were developed with chemiluminescence reagents (Sangon, Shanghai, China).

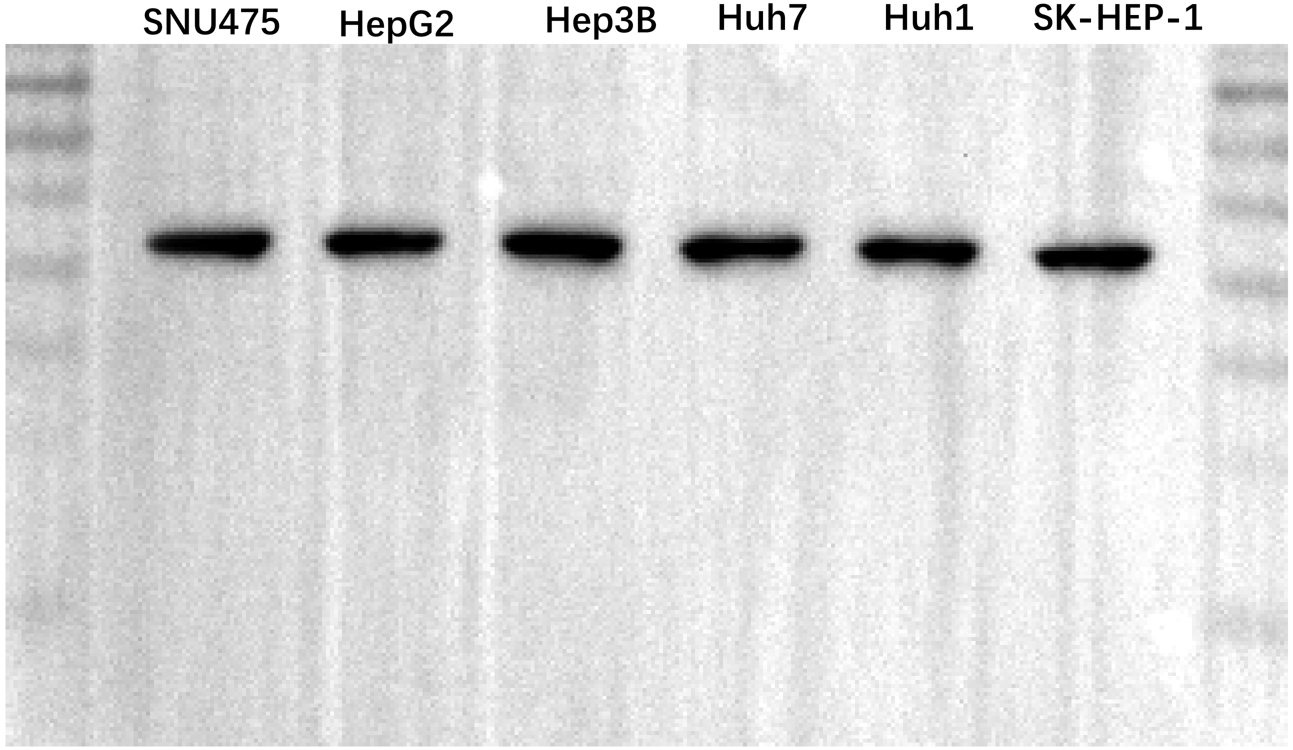

Figure S2 Western blot membrane of GAPDH (35~40 kDa) protein (samples were same as Fig. S1) detected with anti-GAPDH (1:50000; cat no. 60004-1-Ig, Proteintech, Wuhan, China) antibody. Gel-separated proteins were transferred to nitrocellulose membranes (0.2 um pore size; Thermo Fisher Scientific, USA) by wet electroblotting (310 mA, 120 min). Membranes, incubated with a HRP-conjugated Affinipure Goat Anti-Mouse IgG(H+L) (1:3000, Cat no. SA00001-1, Proteintech, Wuhan, China), were developed with chemiluminescence reagents (Sangon, Shanghai, China).

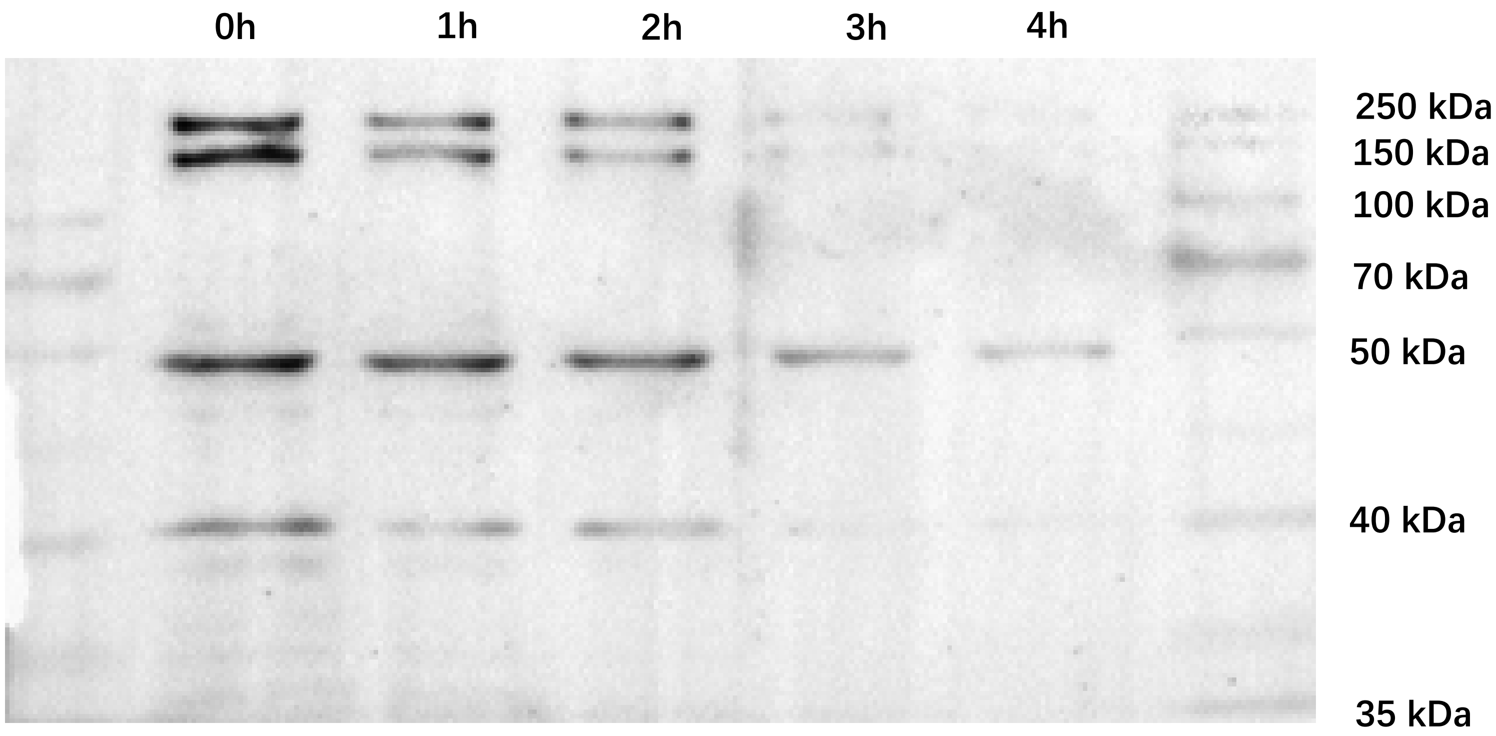

Figure S3 Western blot membrane of XIRP2 (>250 kDa) protein detected with anti-XIRP2 (1:500; cat no. 11896-1-AP, Proteintech, Wuhan, China) antibody in Huh7 cells. Gel-separated proteins were transferred to nitrocellulose membranes (0.2 um pore size; Thermo Fisher Scientific, USA) by wet electroblotting (310 mA, 120 min). Membranes, incubated with a HRP-conjugated Affinipure Goat Anti-Rabbit IgG(H+L) (1:3000, Cat no. SA00001-2, Proteintech, Wuhan, China), were developed with chemiluminescence reagents (Sangon, Shanghai, China).

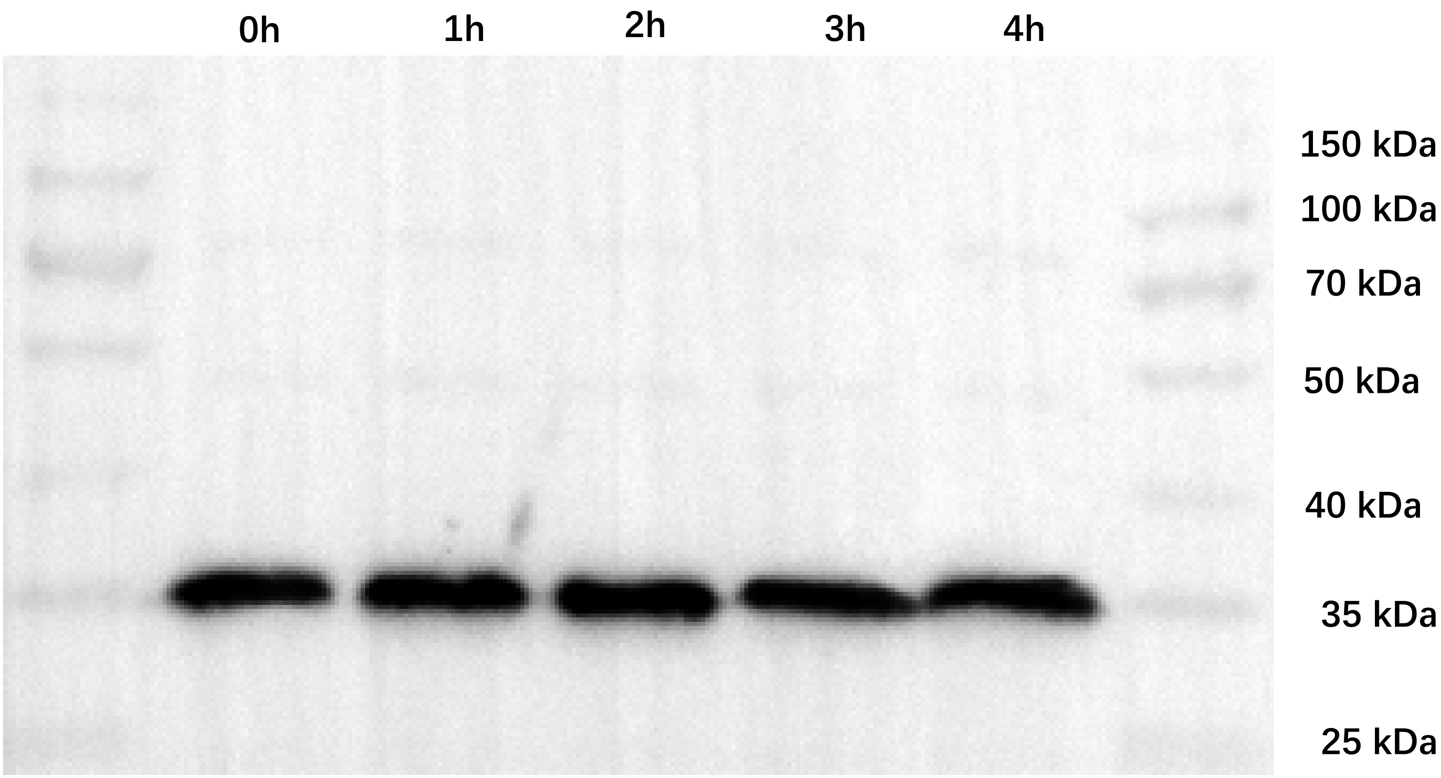

Figure S4 Western blot membrane of GAPDH (35~40 kDa) protein (samples were same as Fig. S3) detected with anti-GAPDH (1:50000; cat no. 60004-1-Ig, Proteintech, Wuhan, China) antibody in Huh7 cells. Gel-separated proteins were transferred to nitrocellulose membranes (0.2 um pore size; Thermo Fisher Scientific, USA) by wet electroblotting (310 mA, 120 min). Membranes, incubated with a HRP-conjugated Affinipure Goat Anti-Mouse IgG(H+L) (1:3000, Cat no. SA00001-1, Proteintech, Wuhan, China), were developed with chemiluminescence reagents (Sangon, Shanghai, China).

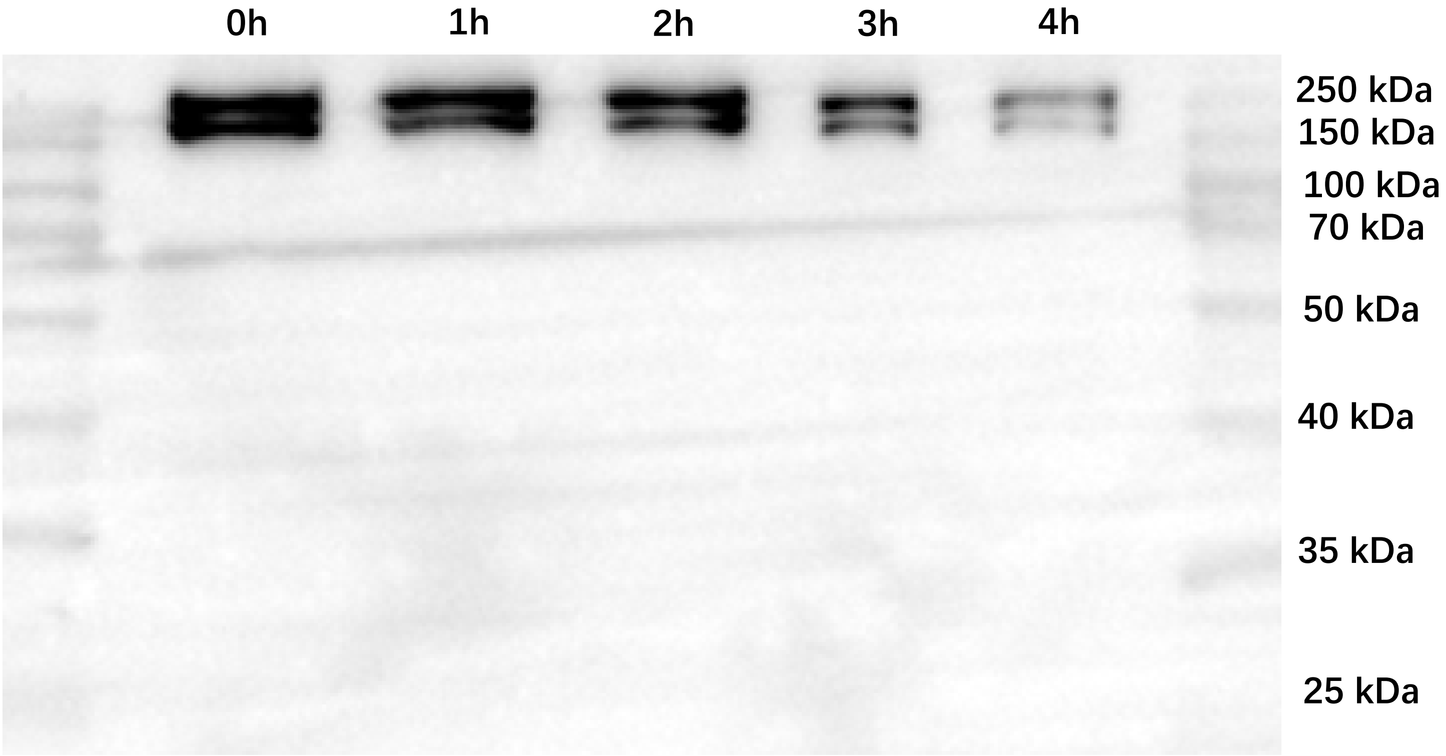

Figure S5 Western blot membrane of XIRP2 (>250 kDa) protein detected with anti-XIRP2 (1:500; cat no. 11896-1-AP, Proteintech, Wuhan, China) antibody in SNU475 cells. Gel-separated proteins were transferred to nitrocellulose membranes (0.2 um pore size; Thermo Fisher Scientific, USA) by wet electroblotting (310 mA, 120 min). Membranes, incubated with a HRP-conjugated Affinipure Goat Anti-Rabbit IgG(H+L) (1:3000, Cat no. SA00001-2, Proteintech, Wuhan, China), were developed with chemiluminescence reagents (Sangon, Shanghai, China).

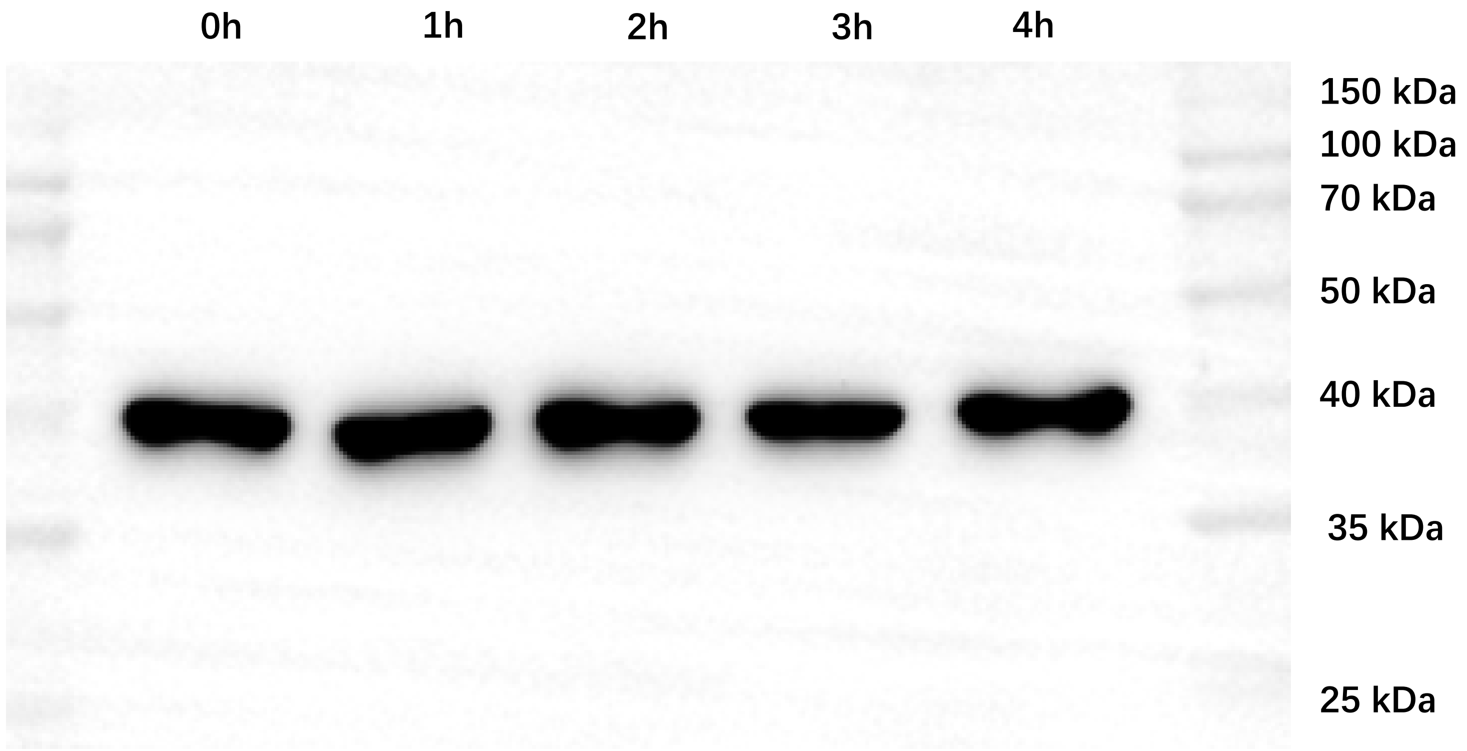

Figure S6 Western blot membrane of GAPDH (35~40 kDa) protein (samples were same as Fig. S5) detected with anti-GAPDH (1:50000; cat no. 60004-1-Ig, Proteintech, Wuhan, China) antibody in SNU475 cells. Gel-separated proteins were transferred to nitrocellulose membranes (0.2 um pore size; Thermo Fisher Scientific, USA) by wet electroblotting (310 mA, 120 min). Membranes, incubated with a HRP-conjugated Affinipure Goat Anti-Mouse IgG(H+L) (1:3000, Cat no. SA00001-1, Proteintech, Wuhan, China), were developed with chemiluminescence reagents (Sangon, Shanghai, China).

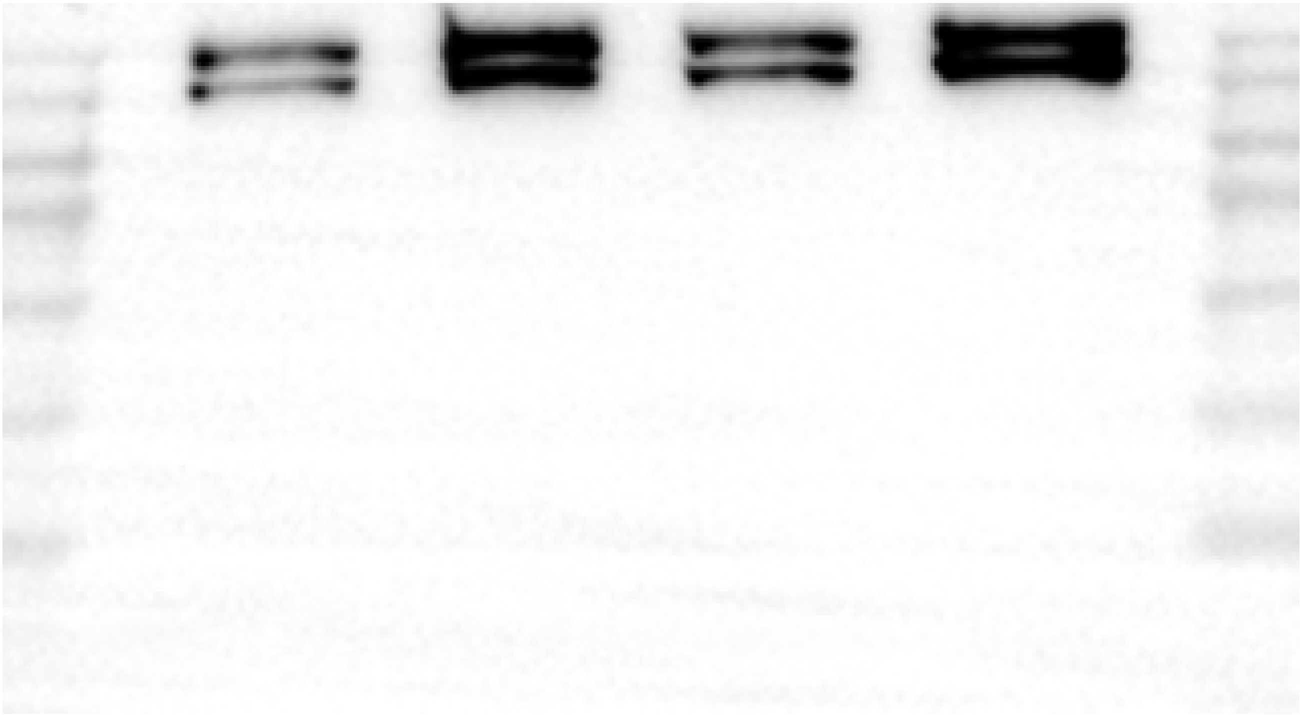

Figure S7 Western blot membrane of XIRP2 (>250 kDa) protein detected with anti-XIRP2 (1:500; cat no. 11896-1-AP, Proteintech, Wuhan, China) antibody in Huh7 and Huh1 cells. Gel-separated proteins were transferred to nitrocellulose membranes (0.2 um pore size; Thermo Fisher Scientific, USA) by wet electroblotting (310 mA, 120 min). Membranes, incubated with a HRP-conjugated Affinipure Goat Anti-Rabbit IgG(H+L) (1:3000, Cat no. SA00001-2, Proteintech, Wuhan, China), were developed with chemiluminescence reagents (Sangon, Shanghai, China).

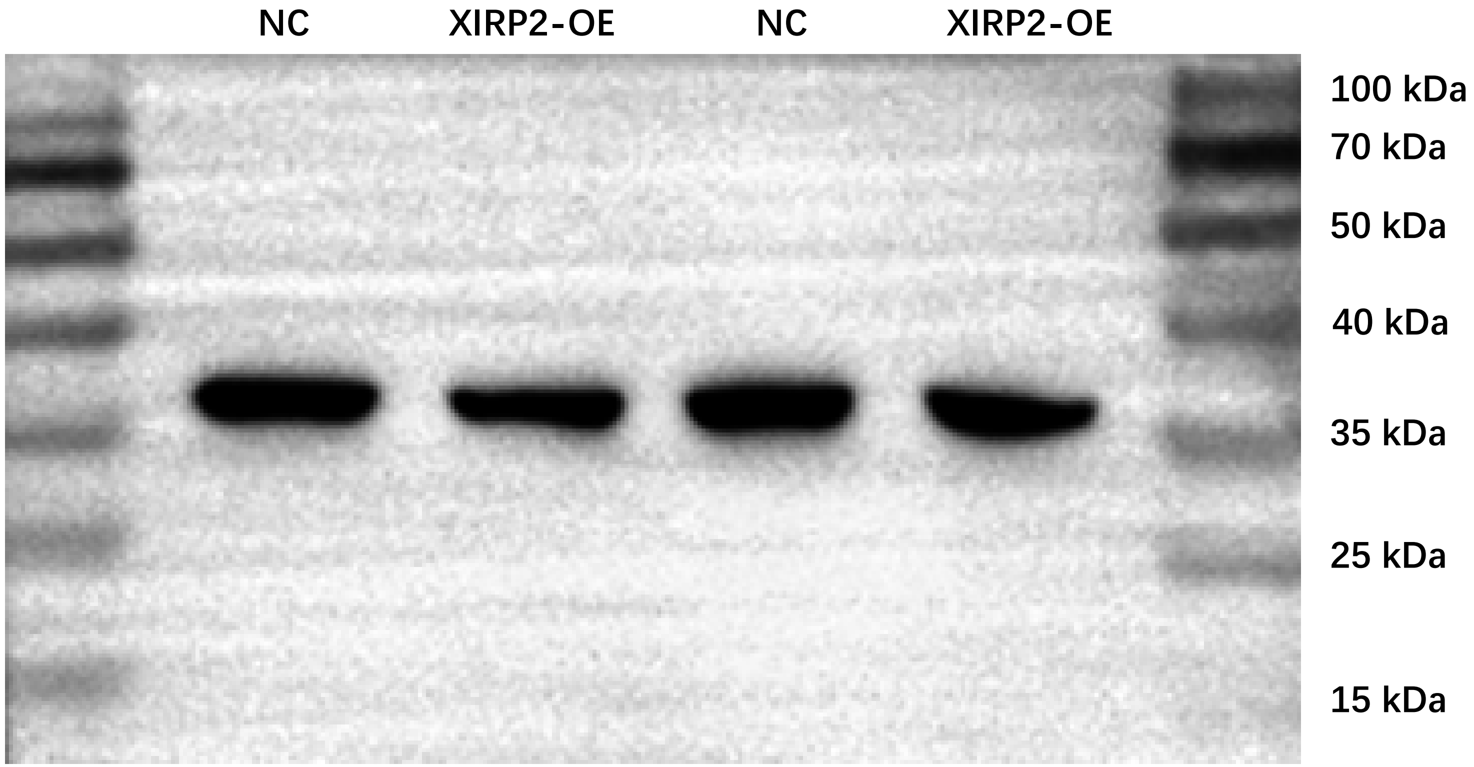

Figure S8 Western blot membrane of GAPDH (35~40 kDa) protein (samples were same as Fig. S7) detected with anti-GAPDH (1:50000; cat no. 60004-1-Ig, Proteintech, Wuhan, China) antibody in Huh7 and Huh1 cells. Gel-separated proteins were transferred to nitrocellulose membranes (0.2 um pore size; Thermo Fisher Scientific, USA) by wet electroblotting (310 mA, 120 min). Membranes, incubated with a HRP-conjugated Affinipure Goat Anti-Mouse IgG(H+L) (1:3000, Cat no. SA00001-1, Proteintech, Wuhan, China), were developed with chemiluminescence reagents (Sangon, Shanghai, China).

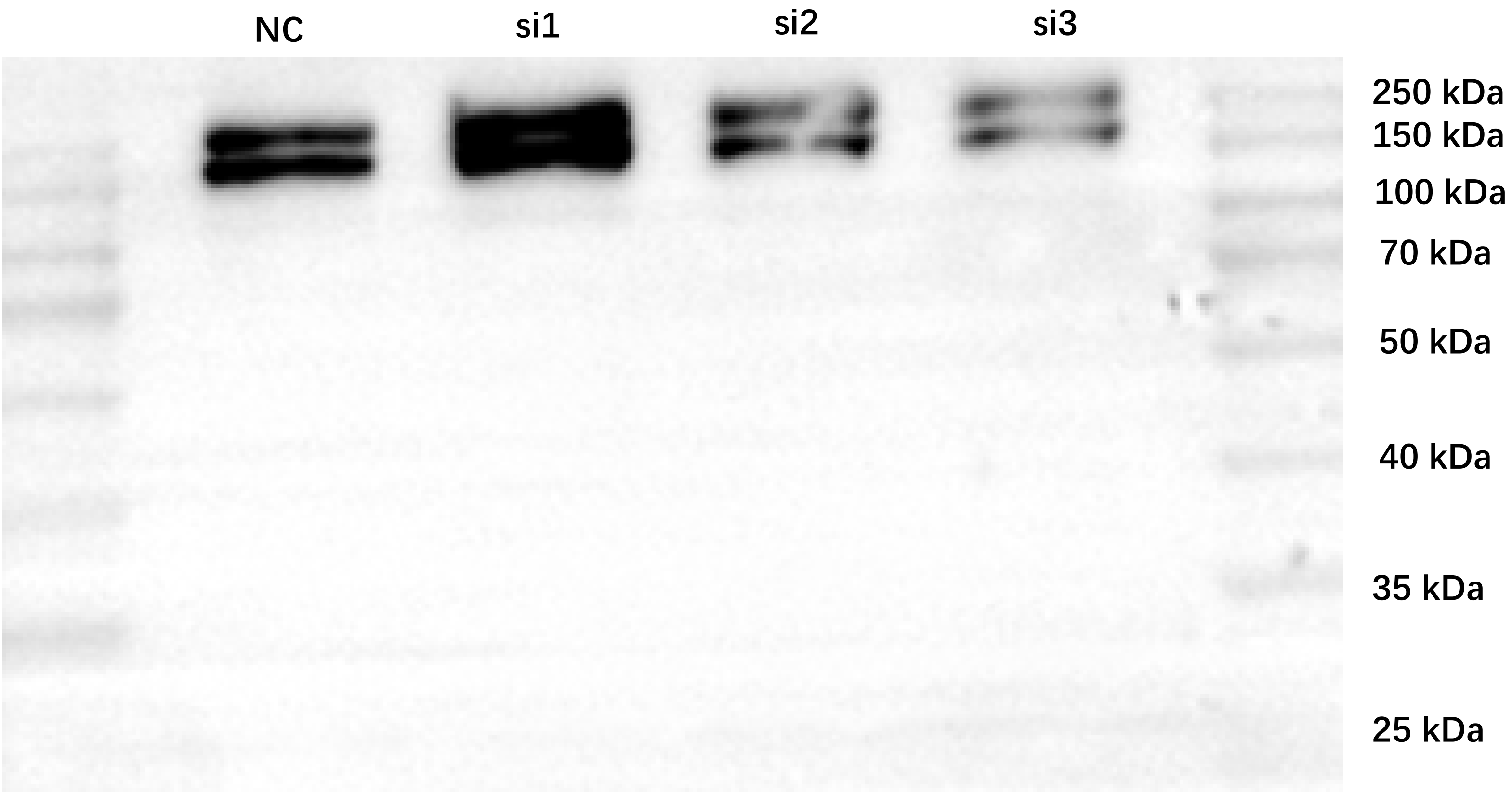

Figure S9 Western blot membrane of XIRP2 (>250 kDa) protein detected with anti-XIRP2 (1:500; cat no. 11896-1-AP, Proteintech, Wuhan, China) antibody in SNU475 cells. Gel-separated proteins were transferred to nitrocellulose membranes (0.2 um pore size; Thermo Fisher Scientific, USA) by wet electroblotting (310 mA, 120 min). Membranes, incubated with a HRP-conjugated Affinipure Goat Anti-Rabbit IgG(H+L) (1:3000, Cat no. SA00001-2, Proteintech, Wuhan, China), were developed with chemiluminescence reagents (Sangon, Shanghai, China).

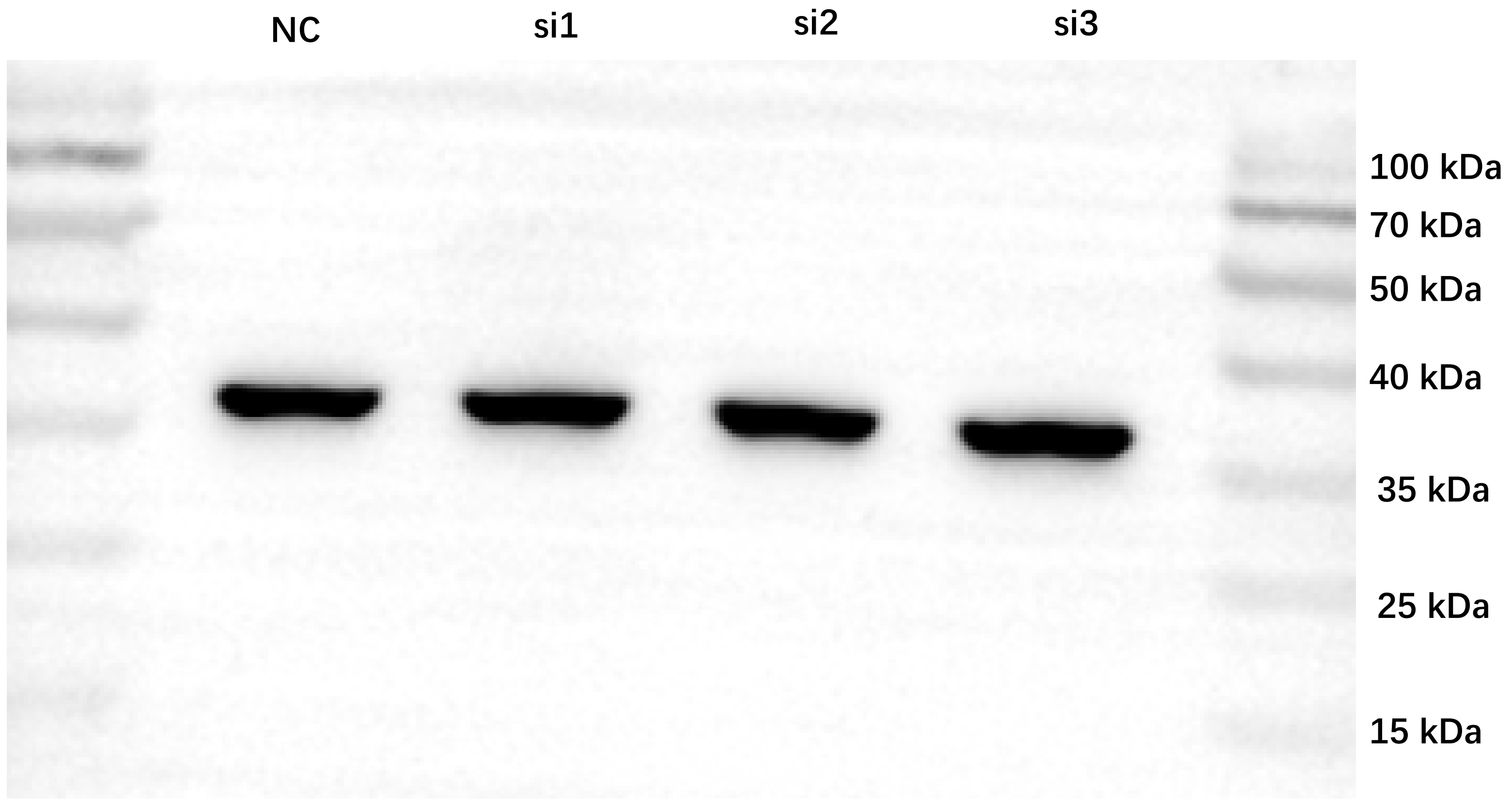

Figure S10 Western blot membrane of GAPDH (35~40 kDa) protein (samples were same as Fig. S9) detected with anti-GAPDH (1:50000; cat no. 60004-1-Ig, Proteintech, Wuhan, China) antibody in SNU475 cells. Gel-separated proteins were transferred to nitrocellulose membranes (0.2 um pore size; Thermo Fisher Scientific, USA) by wet electroblotting (310 mA, 120 min). Membranes, incubated with a HRP-conjugated Affinipure Goat Anti-Mouse IgG(H+L) (1:3000, Cat no. SA00001-1, Proteintech, Wuhan, China), were developed with chemiluminescence reagents (Sangon, Shanghai, China).
